# Commentary: Beauty Requires Thought

**DOI:** 10.3389/fpsyg.2017.01281

**Published:** 2017-08-02

**Authors:** Severi Luoto

**Affiliations:** ^1^English, Drama and Writing Studies, University of Auckland Auckland, New Zealand; ^2^School of Psychology, University of Auckland Auckland, New Zealand

**Keywords:** beauty, cognition, visual processing, aesthetics, reward, consciousness, unconscious processing, physical attractiveness

Brielmann and Pelli (2017) analyzed the relationship between cognitive processing and the appreciation of beauty. They reached the conclusion that thought is a prerequisite for feeling beauty. I argue that the authors misrepresent philosophy, common sense, and cognitive psychology by (a) distinguishing visually mediated beauty from sensory pleasures, and (b) arguing that that distinction is empirically grounded. As I also proceed to demonstrate, cognitive processing is not a prerequisite for the appreciation of beauty, nor have Brielmann and Pelli (2017) provided adequate evidence to plausibly argue to the contrary.

Advances in cognitive science indicate that beauty can be appraised in an astonishingly short period of time. Whatever cognitive processing is evoked by a particular sensory stimulus does not preclude the fact that physical beauty can be appraised quickly and without elaborate cognitive processing. Physical attractiveness can be assessed within an exposure time of 100 ms (Willis and Todorov, 2006) and can result in an affective response indexed by pupil dilation within 500 ms (Finke et al., 2017). What is more, attractiveness judgments made in 100 ms are highly correlated with judgments made without time constraints (Willis and Todorov, 2006). These findings pose a serious challenge to Brielmann and Pelli's argument. Infants as young as 2–3 months look longer at attractive female faces than unattractive ones (Langlois et al., 1987), lending further evidence to the argument that the appreciation of beauty does not require sophisticated cognitive processing (Chatterjee et al., 2009).

As it turns out, the affective component of a visual stimulus can even be appraised without conscious awareness. Strikingly, arousal to sexual stimuli can occur in absence of conscious awareness of the stimulus which evoked it (Ponseti and Bosinski, 2010; Gillath and Collins, 2016). Although the sexual stimuli used in these studies may not align with what Brielmann and Pelli (2017) had in mind when discussing beauty, the finding that visually pleasing stimuli can cause affective and sexual responses even without conscious awareness poses another challenge to their argument (Ponseti and Bosinski, 2010; Gillath and Collins, 2016; cf. Chatterjee et al., 2009). The counterargument—that thought is not a prerequisite for an affective response to visual stimuli—is also supported by the finding that visual exposure to faces from out-group ethnical groups can elicit inter-racial affective bias *outside conscious awareness* (Yuan et al., 2017). Brielmann and Pelli ought to have considered additional experiments, say, on suppressing subjects' conscious awareness (Yang et al., 2014) to more reliably test their hypotheses.

Reviewing work on visual information integration outside of consciousness, Mudrik et al. (2014) found that there is no absolute dependency on consciousness for information integration to occur. The more complex or novel the stimuli, the more necessary consciousness is for information integration (Mudrik et al., 2014). Consciousness enables information integration over extended distances and durations, while also facilitating integration of novel associations over higher semantic levels using multiple modalities (Mudrik et al., 2014). However, there *are* integrative processes that can occur outside of conscious awareness (Mudrik et al., 2014), which provides empirical weight to the claim that conscious awareness or complex cognitive processes are not prerequisites for the appreciation of beauty.

Brielmann and Pelli's (2017) claim that beauty requires thought is based on a dubious interpretation of their experimental results. Even when cognitively distracted, a fair proportion of the subjects rated the experimental stimuli as definitely beautiful. For example, 55% of the participants in Experiment 1B rated the ‘self-selected beautiful’ stimulus as definitely beautiful despite the experimental interference of cognitive distraction (Brielmann and Pelli, 2017). Contrary to the authors' conclusion, this finding could be interpreted in such a way that the appreciation of beauty does *not* require thought: 55% of the subjects, after all, experienced beauty despite cognitive distraction. The experimental data provided by Brielmann and Pelli (2017) is simply inadequate to conclude that “the pleasure associated with feeling beauty requires thought.”

Separating visually mediated beauty from sensory pleasures is incongruent with common sense, cognitive psychology, and with Immanuel Kant—he did not claim that in the works cited by Brielmann and Pelli. Beauty of the kind represented in the visual stimuli that Brielmann and Pelli (2017) used is appraised visually, making it as much a *sensory pleasure* as the tactile and gustatory stimuli provided by the teddy bear and the candy used in their experiments. There are no grounds to separate visual pleasures from other sensory pleasures—all are ways in which organisms acquire information from the external world, all are ways in which organisms become incentivized toward stimuli that, in the aggregate, tend to be evolutionarily useful, or tend to tap onto psychological mechanisms which make organisms appraise them as such (Sperber and Hirschfeld, 2004; Berridge and Kringelbach, 2013).

Besides the sensory component, appreciation of beauty can consist of two other components: an emotional response evoked by the beautiful stimulus, and a cognitive evaluation of the sets of meanings associated with the stimulus (Bromberger et al., 2011; Chatterjee, 2011). Interestingly, damage to the right hemisphere can impair evaluation of the content-conceptual qualities of art without affecting preference for the art (Bromberger et al., 2011). It therefore seems necessary to subdivide appreciation of beauty into at least three different components: sensory, cognitive, and emotional (Figure 1) (Bromberger et al., 2011; Chatterjee, 2011). Brielmann and Pelli's (2017) experimental intervention might have impacted the cognitive component, but since beauty is not reliant on thought, appreciating beauty is still possible when the cognitive component has been impaired (cf. Bromberger et al., 2011).

**Figure 1:**
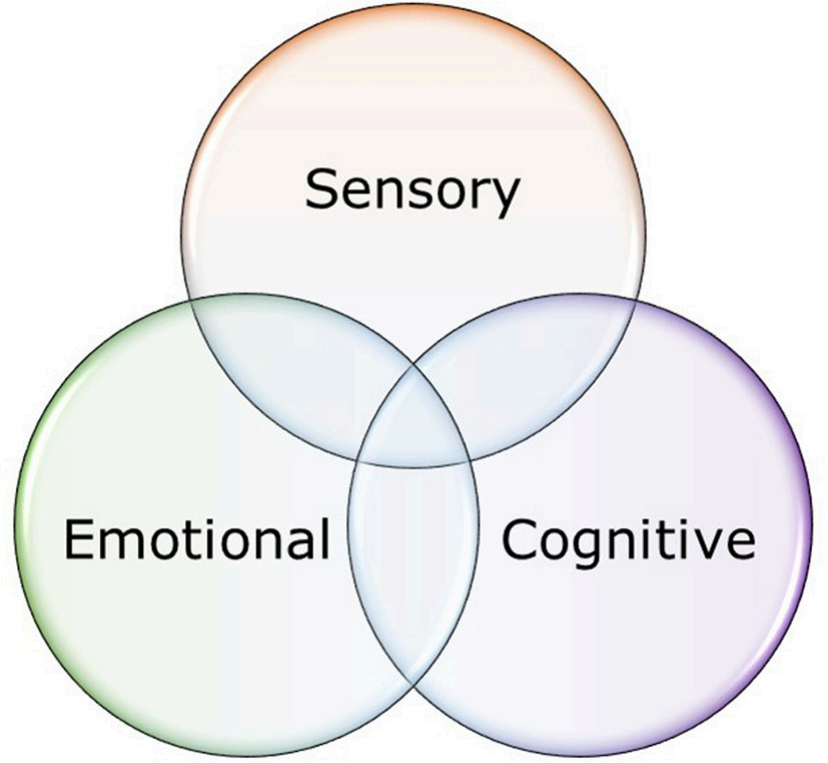
Three primary constituents of beauty response. Any two can be removed without completely incapacitating a person's ability to appreciate beauty (cf. Bromberger et al., 2011; Nadal, 2013).

In conclusion, an aesthetic judgment of beauty is firmly grounded in sensory processes (Jacobsen, 2006), and there are no empirical grounds to cleave visually mediated appreciation of beauty from sensory pleasures. Beauty *can* be amplified by cognitive processes (Vessel et al., 2012)—such as integration with novel associations, integration over higher semantic levels, or integration over multiple modalities (Mudrik et al., 2014)—processes which can be particularly important for the experience of art (Nadal, 2013). Yet as demonstrated above, elaborate cognitive processes are by no means a prerequisite for the appreciation of beauty.

## Author contributions

The author confirms being the sole contributor of this work and approved it for publication.

### Conflict of interest statement

The author declares that the research was conducted in the absence of any commercial or financial relationships that could be construed as a potential conflict of interest.
